# Radiofrequency Ablation of Parathyroid Glands to Treat a Patient With Hypercalcemia Caused by a Novel Inactivating Mutation in *CaSR*

**DOI:** 10.3389/fendo.2021.743517

**Published:** 2022-01-14

**Authors:** Yu Hao, Zhikai Lei, Nanjing Shi, Lingying Yu, Weiqin Ji, Xianfeng Zhang

**Affiliations:** ^1^ Department of Endocrinology, Affiliated Hangzhou First People’s Hospital, Zhejiang University School of Medicine, Hangzhou, China; ^2^ Department of Ultrasound, Affiliated Hangzhou First People’s Hospital, Zhejiang University School of Medicine, Hangzhou, China

**Keywords:** calcium-sensing receptor, hypocalciuric hypercalcemia, inactivating mutation, parathyroid gland, radiofrequency ablation

## Abstract

**Objective:**

We identified a novel inactivating mutation in the calcium-sensing receptor (CaSR) gene in a patient with refractory hypocalciuric hypercalcemia and analyzed its function. The effectiveness of radiofrequency ablation of the parathyroid glands to treat hypercalcemia caused by this mutation was explored.

**Methods:**

Clinical data of patients before and after radiofrequency ablation were retrospectively analyzed. The CaSR mutation (D99N) found in the patient was studied in cell lines. HEK-293 cells were transfected with plasmids containing wild-type (WT) or mutant CaSR genes (D99N and W718X). Expression levels of the respective CaSR proteins were measured, and their functions were assessed by examining the effect of NPS R-568 (a CaSR agonist) on intracellular Ca^2+^ oscillations and that of exogenous parathyroid hormone (PTH) on intracellular cyclic adenosine monophosphate (cAMP) levels.

**Results:**

The effectiveness of pharmacological treatment was poor, whereas radiofrequency ablation of the parathyroid glands resulted in controlled blood calcium and PTH levels in the patient. In cell lines, upon NPS R-568 administration, the amplitude of intracellular Ca^2+^ oscillations in the D99N group was lower than that in the WT group and higher than that in the W718X group. Upon administration of PTH, intracellular cAMP levels in the D99N group were higher than those in the WT group and lower than those in the W718X group.

**Conclusion:**

The homozygous mutation D99N reduced CaSR activity and caused more severe hypocalciuric hypercalcemia. For patients with this type of hypercalcemia and poor response to pharmacological treatments, radiofrequency ablation of the parathyroid glands may be a suitable treatment option.

## Introduction

Calcium-sensing receptor (CaSR) is a member of the G protein-coupled receptor superfamily. It is expressed in various body tissues, including the parathyroid glands, kidneys, bones, gastrointestinal tract, livers, central nervous system, breasts, and pituitary gland. Specifically, CaSR is highly expressed in the parathyroid glands and kidney (1). The primary function of CaSR is to sense increases in extracellular Ca^2+^ concentrations and consequently inhibit the secretion of parathyroid hormone (PTH) from the parathyroid glands, thereby promoting Ca^2+^ excretion by the renal tubules, which helps to maintain the blood calcium concentration.

In the presence of increased Ca^2+^ concentrations, CaSR may inhibit the activity of adenylate cyclase *via* Gi protein– or Gq/11 protein–dependent intracellular Ca^2+^ mobilization, leading to decreased PTH secretion (2). Furthermore, CaSR activation inhibits the enhanced renal tubular reabsorption of divalent cations (including Ca^2+^ and Mg^2+^) mediated by increased intracellular cyclic adenosine monophosphate (cAMP) induced by PTH (3). Intracellular Ca^2+^ and other second messengers involving in various types of cellular reactions is closely regulated by CaSR. Extracellular Ca^2+^ is the principal agonist of CaSR, aliphatic and aromatic L-amino acids are considered allosteric activators, while extracellular phosphate inhibits CaSR activity (4, 5). Structural framework for CaSR activation by Ca^2+^ and other ligands has disclosed recently (6).

Mutation in the CaSR gene can lead to functional changes in the receptor protein and alter the sensitivity of cells to Ca^2+^and causes a wide variety of human diseases (7). Activing mutations of CaSR lead to autosomal dominant hypocalemia (ADH), while heterozygous or homozygous inactivating mutations of CaSR lead to different extents of hypercalcemia accompanied by hypocalciuria, such as familial hypocalciuric hypercalcemia1 (FHH1) and neonatal severe hyperparathyroidism (NSHPT). Calcimimetics, such as cinacalcet and NPS R-568, bind to the transmembrane domain of CaSR and increase its sensitivity to Ca^2+^ in an allosteric manner, thereby enhancing signal transduction and function (8, 9).

In this study, a novel mutation in the CaSR gene in a patient with hypocalciuric hypercalcemia was identified, and functional analysis was performed to provide a basis for diagnosis and treatment. The effectiveness of radiofrequency ablation of the parathyroid glands was also studied. To the best of our knowledge, this is the first case of parathyroid radiofrequency ablation for hypercalcemia associated with a CaSR gene mutation.

## Material and Methods

### Clinical Data and CaSR Mutation Analysis of the Patient

The clinical data of a single patient with hypocalciuric hypercalcemia were retrospectively analyzed before and after radiofrequency ablation. Informed consent was obtained from the patient and his parents before genomic testing. The study was approved by the ethics committee of Affiliated Hangzhou First People’s Hospital, Zhejiang University School of Medicine, Hangzhou, Zhejiang province, China and was performed in accordance with the ethical standards as laid down in the 1964 Declaration of Helsinki and its later amendments or comparable ethical standards. Genomic DNA was extracted from the peripheral blood using an Invitrogen genomic DNA extraction kit (Invitrogen, Carlsbad, CA, USA). Standard polymerase chain reaction was carried out to amplify the CaSR gene in a 25-µL reaction mixture containing 1 µL DNA, targeted primers, 0.5 µL dNTPs, and 0.25 µL Taq polymerase (Thermo Fisher Scientific, Waltham, MA, USA; catalogue no.10342020), using the ABI Gene Amp PCR System 9700 (Applied Biosystems, Foster City, CA, USA). The amplified products were analyzed by gel electrophoresis, purified, and sequenced using a 3730XL DNA Sequencer (Applied Biosystems). Sequence analysis results were analyzed using Variant Reporter software v1.0.

### Radiofrequency Ablation of Parathyroid Glands

Ablation was performed by an experienced ultrasound interventional doctor using a VIVA RF Generator (VRS01, STARmed, Gyeonggi-do, South Korea). The patient was kept in the supine position with the neck fully exposed, and the left inferior parathyroid gland was explored and located. After routine disinfection, a liquid-isolating zone was set up by injecting 10mL 2% lidocaine diluted in normal saline (1:1) into the space between the left inferior parathyroid gland and the surrounding tissue under real-time ultrasound guidance. The radiofrequency needle was inserted into the parathyroid gland, and ablation was started at the power 35 W. Until the high echo completely covered the left inferior parathyroid gland, the radiofrequency needle was pulled out after completing the routine radiofrequency therapy procedure. The left superior parathyroid gland was treated in the same manner. Three days later, the patient had no obvious discomfort such as hoarseness, and radiofrequency ablation of the right inferior parathyroid gland was performed.

### Recombinant Plasmid Construction and Validation

Overlap extension was performed on the human wild-type (WT) CaSR expression vector pcDNA3.1-CaSR-WT (Synbio Technologies, Suzhou, China) to introduce point mutations in the constructs pcDNA3.1-CaSR D99N (G→A at 295 Exon3) and pcDNA3.1-CaSR W718X. The W718X mutation is a nonsense mutation that changes nucleotide 2153 in the coding region from G to A. This mutation results in a change from a tryptophan-coding codon to a stop codon, leading to the early termination of protein synthesis, thereby generating a protein lacking the last four transmembrane domains and intracellular tail. All expression plasmids were sequenced directly with an ABI 3730XL Genetic Analyzer (Applied Biosystems).

### Cell Culture and Transfection

HEK-293 cells were cultured in Dulbecco’s Modified Eagle’s Medium (GIBCO, Grand Island, NY, USA) containing 10% fetal bovine serum, in a 37°C incubator with 5% CO_2_. Routinely, cultured cells in the log phase were seeded into six-well plates at a density of 5 × 10^5^ cells/well and cultured for 24 h. A total of 5 μg of plasmid and 3.75 μL of Lipofectamine 3000 (Invitrogen) were added to 200 μL of Opti-MEM (GIBCO). The solution was mixed and added to the corresponding cell-culture wells and incubated for 48 h for transfection. Cells transfected with the corresponding plasmids were divided into four groups: blank (empty plasmid), CaSR-WT, CaSR-D99N, and CaSR-W718X.

### Immunofluorescence Study

Immunofluorescence was used to detect the expression of WT and mutated CaSRs (D99N and W718X) in transfected cells. The transfected HEK-293 cells were transferred into 12-well plates (Corning, Inc., Corning, NY, USA) containing sterile glass coverslips. After approximately 48 h, the cells were immobilized in 4% paraformaldehyde (Sigma, St. Louis, MO, USA) and treated with phosphate buffered saline (PBS, GIBCO) containing 0.2% Triton X-100 (Sigma) for permeabilization. After being incubated with 5% bovine serum albumin, the cells were again incubated over night with mouse monoclonal antibody against human CaSR aa200-300 (Abcam, Cambridge, UK; ab19347) at a dilution of 1:100. The cells were then washed three times for 5 min each and then incubated for 1 h at room temperature with anti-mouse IgG antibody conjugated to Alexa Fluor 488 secondary antibody (Abcam; ab150077). Fluorescence images were obtained using an Olympus FLUOVIEW FV1000 laser confocal microscope (Tokyo, Japan).

### Western Blot Assay

After cell transfection, the supernatant was discarded, and the cells were washed twice with PBS before lysing with lysis buffer (20 mM Tris HCl, 150 mM NaCl, 1% NP-40). After centrifugation, the protein concentration in the supernatant was determined using a BCA Protein Assay Kit (Beyotime, Shanghai, China). An aliquot of protein was denatured by boiling in a 100°C water bath for 5 min. SDS-polyacrylamide gel electrophoresis of the denatured proteins (20 μg protein per well) was performed using an 8% separating gel and 5% stacking gel. The separated proteins were transferred onto a polyvinylidene fluoride membrane, which was blocked before incubating with mouse anti-CaSR ab19347 antibody (Abcam) followed by horseradish peroxidase-conjugated anti-mouse IgG antibody (Thermo Fisher Scientific; Product # 31430). Enhanced chemiluminescent reagent (Thermo Fisher Scientific) was added, and the fluorescent signals were imaged using a Chemiluminescence Fluorescence Image Analyzer (FUJIFILM, Osaka, Japan).

### Fluorescent Probes for Measuring Intracellular Ca^2+^ Concentration

The AM esters of the fluorescent probesFluo-3 (Invitrogen; F23915) and FuraRed (Invitrogen; F3021) were separately diluted with Ca^2+^-free extracellular solution (135 mM NaCl, 2 mM KCl, 2 mM MgCl_2_, 10 mM HEPES, 22.2mM glucose; pH 7.4) to a working concentration of 5 μM and 15 μM, respectively. After 24 h of cell subculture, the medium was discarded, and the cells were washed with PBS 3 times. The Fluo-3 and FuraRed working solutions were separately added to the cells, which were then incubated at 37°C for 40 min. The fluorescent probe was discarded and replaced with new Ca^2+^-free extracellular solution. The cells were incubated at 37°C for 20 min to ensure that the AM ester was fully hydrolyzed. Cells were examined using an Olympus FLUOVIEW FV1000 laser confocal microscope, and cells exhibiting good adherence, extended morphology, and bright fluorescence intensity were selected. The scanning conditions were set at an excitation wavelength of 488 nm, and emission fluorescence at 530/30nm (Fluo-3) and 610/20nm (Fura Red) was recorded. One image was collected every 7 s for approximately 25 min. After the fluorescence intensity curve stabilized, NPS R-568 (final concentrations 10 μM, Sigma) was added to each group of cells; after the curve stabilized again, ATP (final concentrations 100 μM, Sigma) was added, and the experiment was continued until the curve had stabilized again. The ratios of the relative peak fluorescence values after NPS R-568 and ATP addition were analyzed.

### ELISA Measurement of Intracellular cAMP Levels

Intracellular cAMP levels were determined in the different cell groups after dimethyl sulfoxide (control) or PTH (30 nM, Sigma) was added to the transfected cells and incubation for 6 h. A cAMP ELISA kit (Enzo Life Sciences, Farmingdale, NY, USA) was used according to the manufacturer’s protocol.

### Statistical Analysis

Statistical analysis was performed with SPSS software v14.0 (SPSS, Inc., Chicago, IL, USA). Values are reported as the mean ± standard error of the mean. A *t-*test was used to compare differences in intracellular Ca^2+^ concentrations and cAMP levels. P < 0.05 was considered to indicate statistically significant results.

## Results

### Patient Clinical Data Upon Diagnosis and Treatment

The patient was a 40-year-old male. A physical examination seven years prior revealed elevated blood calcium but no apparent discomfort. His blood calcium remained high in several subsequent follow-ups, with the highest level reaching 4.01 mmol/L. He had a history of hypertension, type 2 diabetes mellitus, and fatty liver disease. The control of blood pressure (*via* amlodipine tablets) and blood glucose (*via* diet) was acceptable. The patient’s paternal and maternal grandmothers were half-sisters. The patient’s mother had mild hypocalciuric hypercalcemia (calcium in 2.8 mmol/L), and any other family history of parathyroid disease or a medication history of thiazide diuretics, lithium preparations, and calcium-containing antacids was denied. The patient’s height and weight were 165 cm and 75 kg, respectively. No physical anomalies were found upon examination.

**Figure 1:**
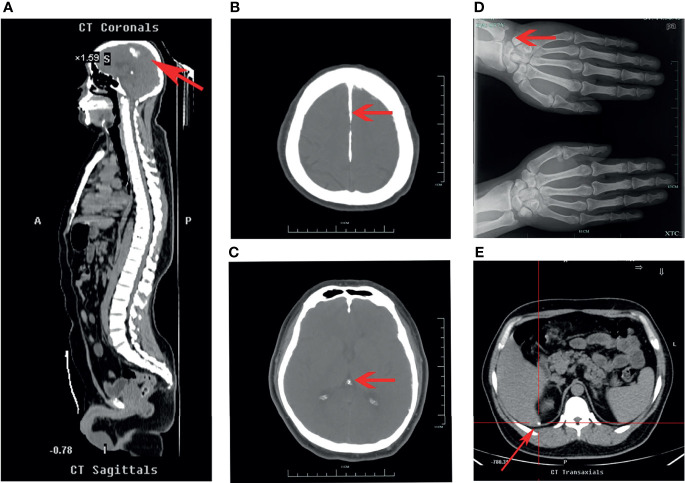
Upper half of body CT, head CT, and X-ray images of the both hands of the patient. **(A, B)** Upper half of body CT and head CT scans, respectively. Red arrows both indicate calcification of cerebral falx. **(C)** Head CT scan. Red arrow indicates calcification of tentorium cerebelli. **(D)** X-ray of both hands. Red arrow indicates calcium deposition. **(E)** Abdominal CT scan. Red arrow indicates calcification in the subcapsular region of the posterior segment of the right liver lobe.

The lab results included high blood calcium, low urine calcium, and abnormal PTH levels (**Table 1**). No abnormalities were observed upon parathyroid ultrasound, and no tumors were detected on emission computed tomography or positron emission tomography–computed tomography (CT) of the parathyroid glands. A cranial CT revealed evident calcification of the falx cerebri and left tentorium cerebelli (**Figures 1A–C**). X-ray images of both hands revealed a change in calcium deposition around the distal ulna (**Figure 1D**). Abdominal CT revealed calcification in the subcapsular region of the posterior segment of the right liver lobe (**Figures 1E**), and B-mode ultrasound of the urinary system showed no evident abnormalities.

**Table 1:** Key patient clinical data.

	Pre-July 2019 report	July 2019 report	After radiofrequency ablation report
**Calcium**	3.69 mmol/L	3.13 mmol/L	2.0–2.3 mmol/L
**Phosphorus**	0.8 mmol/L	1.03 mmol/L	
**PTH**	101 pg/mL	99 pg/mL	9–10 pg/mL
**Creatinine**	76 µmol/L	87 µmol/L	
**Albumin**	46 g/L	42 g/L	
**24-h Urine output**	2800 mL/24 h	3250 mL/24 h	
**24-h Urine calcium excretion**	1.28 mmol (51.5 mg)/24 h	0.68 mmol (2.72 mg)/24 h	
**24-h Urine creatinine excretion**	4278 µmol/24 h	8089 µmol/24 h	
**Urinary calcium to creatinine-ratio**	0.006	0.002	
**25-Hydroxyvitamin D-25**	25 ng/mL		

**Figure 2:**
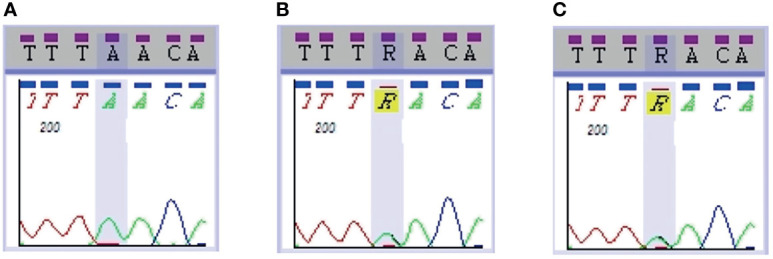
CaSR sequencing results in the patient and parents. Sequencing of the CaSR gene revealed that the patient **(A)** harbored a 295G>A homozygous mutation (D99N), whereas both parents **(B, C)** were R (A/G) heterozygous at the same position.

Gene sequencing results revealed that the patient harbored a G→A mutation at position 295 of exon 3 in the CaSR gene that resulted in an A/A homozygous mutation of CaSR D99N (**Figures 2A**). Sequencing of the CaSR gene of both parents revealed that they both were R (A/G) heterozygous at this position (**Figures 2B, C**).

In a preliminary diagnosis, it was concluded that the patient’s hypercalcemia was caused by an inactivating CaSR mutation based on the clinical lab results and sequence analysis of hypercalcemia-related genes. However, the patient did not respond to treatments with diuretics, calcitonin, or bisphosphonates. Cinacalcet was subsequently administered with initiated dose of 25mg per day, and the patient’s blood calcium levels decreased but was still above normal range. The patient followed a low-calcium diet, drank around 3 L of water daily, and gradually increased cinacalcet dose to 100mg per day. For nearly 4 years, the patient’s blood calcium was maintained at 3.0–3.3 mmol/L and PTH at 70 pg/mL. Owing to the heavy financial burden and poor effectiveness of the calcium-lowering treatments, the patient was admitted to our department in July 2019 for further treatment.

**Figure 3:**
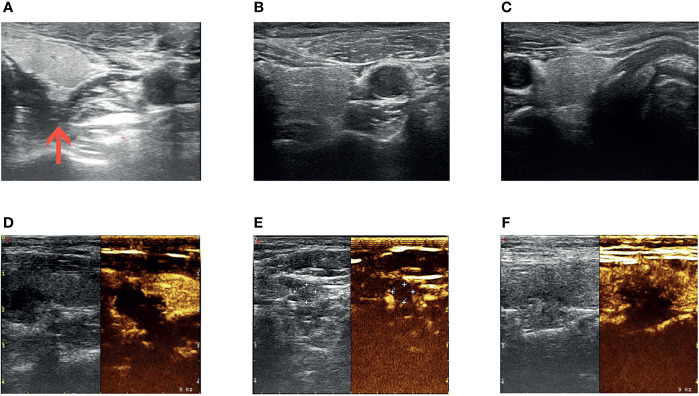
Radiofrequency ablation of the parathyroid glands. Ultrasound images of the left superior **(A)**, left inferior **(B)**, and right superior **(C)** parathyroid glands before radiofrequency ablation. Ultrasound images (left) and ultrasound contrast images (right) of left superior **(D)**, left inferior **(E)**, and right superior **(F)** parathyroid glands after radiofrequency ablation. Red arrow in **(A)** shows that intraoperative saline and lidocaine mixture were used for local anesthesia and isolation of the left superior parathyroid glands and peripheral tissues.

Parathyroid ultrasound displayed echoes from three parathyroid-like structures, although the ultrasound could not confirm the right inferior parathyroid gland (**Figures 3A**–**C**). No hyperparathyroidism was identified on parathyroid imaging. The decision to perform radiofrequency ablation was arrived at after multidisciplinary discussions, complete examination, and ruling out contraindications. Ultrasound-guided radiofrequency ablation of the left inferior and bilateral superior parathyroid glands was performed during several sessions between August and September 2019. After ablation, the parathyroid gland showed very low echo and filling defects (**Figures 3D**–**F**). After the ablation sessions, the patient exhibited hypoparathyroidism, calcitriol (0.25–0.5 µg per day) was administered based on the blood calcium levels. During follow-up examinations after surgery until April 2020, the patient’s blood calcium was maintained at 2.0–2.3 mmol/L, and the blood PTH was maintained at 9–10 pg/mL (**Table 1**).

### Expression and Functional Status of CaSR Protein

**Figure 4:**
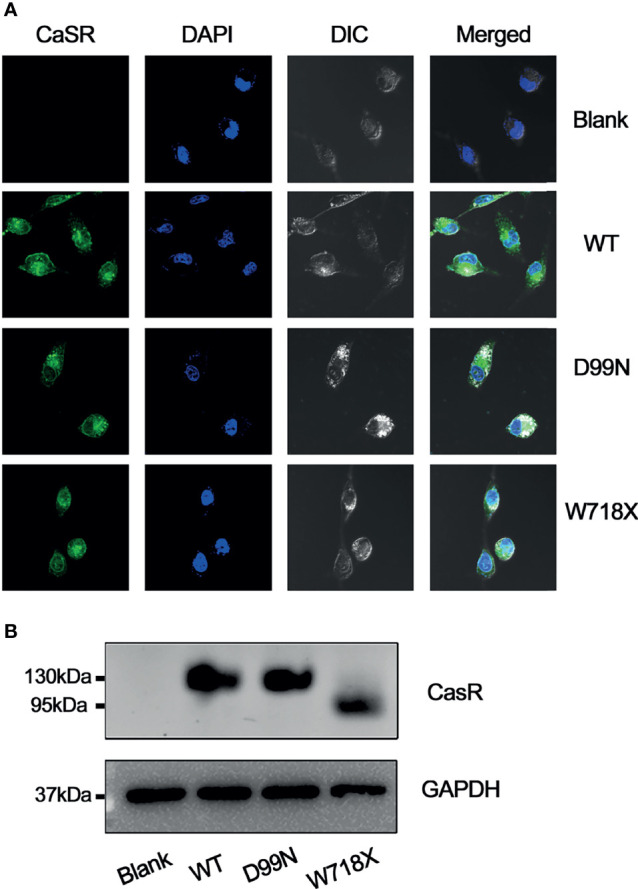
Analysis of CaSR protein expression. **(A)** Expression of CaSR in blank, WT, D99N, and W718X HEK-293 cells was analyzed by immunofluorescence confocal microscopy (1000X) following incubation with a monoclonal anti-CaSR antibody. Green fluorescence indicates CaSR and blue fluorescence (DAPI) indicates the cell nucleus. **(B)** Western blot analysis. Proteins extracted from untransfected HepG2 cells (blank) and HepG2 cells transfected with human wild-type CaSR (WT) or mutant CaSR (D99N and W718X) were subjected to SDS-polyacrylamide gel electrophoresis, western blotting, and visualization using an anti-CaSR monoclonal antibody. GAPDH was used as a loading control.

After transfection, the expression of CaSR protein in each group was examined by immunofluorescence confocal microscopy and western blotting (**Figure 4**). Confocal immunofluorescence showed that CaSR protein was expressed in the cells of each group except the blank group (**Figure 4A**), which is consistent with results of western blotting (**Figure 4B**). Western blotting revealed no expression of CaSR protein in the blank group, whereas the WT and D99N groups showed a 130-kDa protein corresponding to the known molecular weight of the complete CaSR protein. The W718X group expressed a prematurely terminated, smaller protein fragment of 80 kDa (**Figure 4B**).

**Figure 5:**
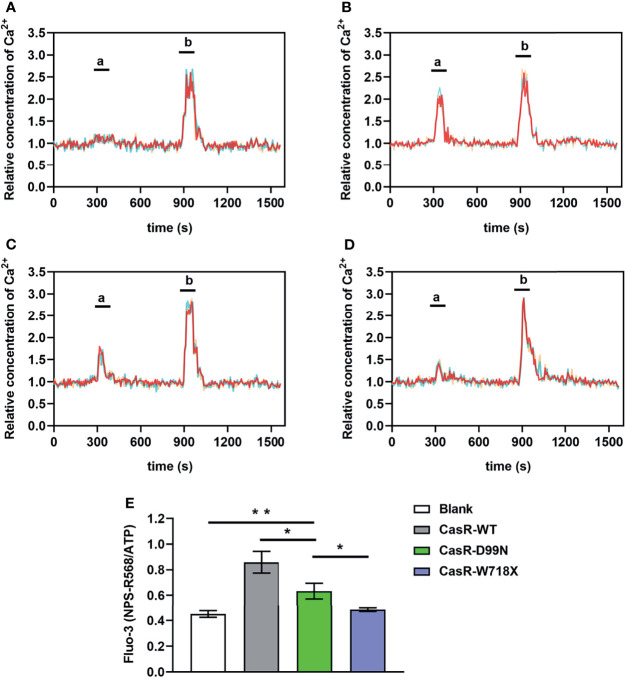
Detection of intracellular calcium concentration using co-loadedFluo-3andFura Redprobes. **(A–D)** Changes in the ratio of Fluo-3/Fura Red fluorescence in response to consecutive stimulation with 10 μM calcimimetics NPS R-568; peak ‘a’) and 100 μM ATP (peak ‘b’) were used as a measure of the intracellular Ca^2+^ concentration in the blank **(A)**, CaSR-WT **(B)**, CaSR-D99N **(C)**, and CaSR-W718X **(D)** groups. **(E)** Relative fluorescence values of NPS R-568/ATP (peak ‘a’/peak ‘b’ in panels **A–D**) in each group, reflecting the Ca^2+^ concentration. Data were obtained from12 wells for each cell type and given as means ± S.E. Statistical significance was determined by Student’s test. *P < 0.05, **P < 0.01. Three separated tests were performed and both inter- and intra- assay coefficients of variability was less than 10%.

The Fluo-3 and Fura Red double labeled fluorescent probe was used to determine intracellular Ca^2+^ concentration, which was represented by the change in Fluo-3/Fura Red ratio (Fluo-3 Ratio). We found that the intracellular Ca^2+^ concentration of the blank and W718X groups did not change significantly after adding 10 μM NPS R-568 at 5 min but increased rapidly and significantly in the WT and D99N groups, however the increase in D99N group was much lower than that in WT group(**Figures 5A**–**D**). At 15 min, there was a transient peak in the intracellular Ca^2+^ concentration in all four groups in response to ATP addition (100 μM). **Figure 5E** shows that the intracellular Ca^2+^ oscillations (Fluo-3ratio of NPS R-568/ATP) in the D99N group were significantly lower than those in the WT group (P < 0.05), and higher than those in the blank (P < 0.01) and W718X groups (P < 0.01).

**Figure 6:**
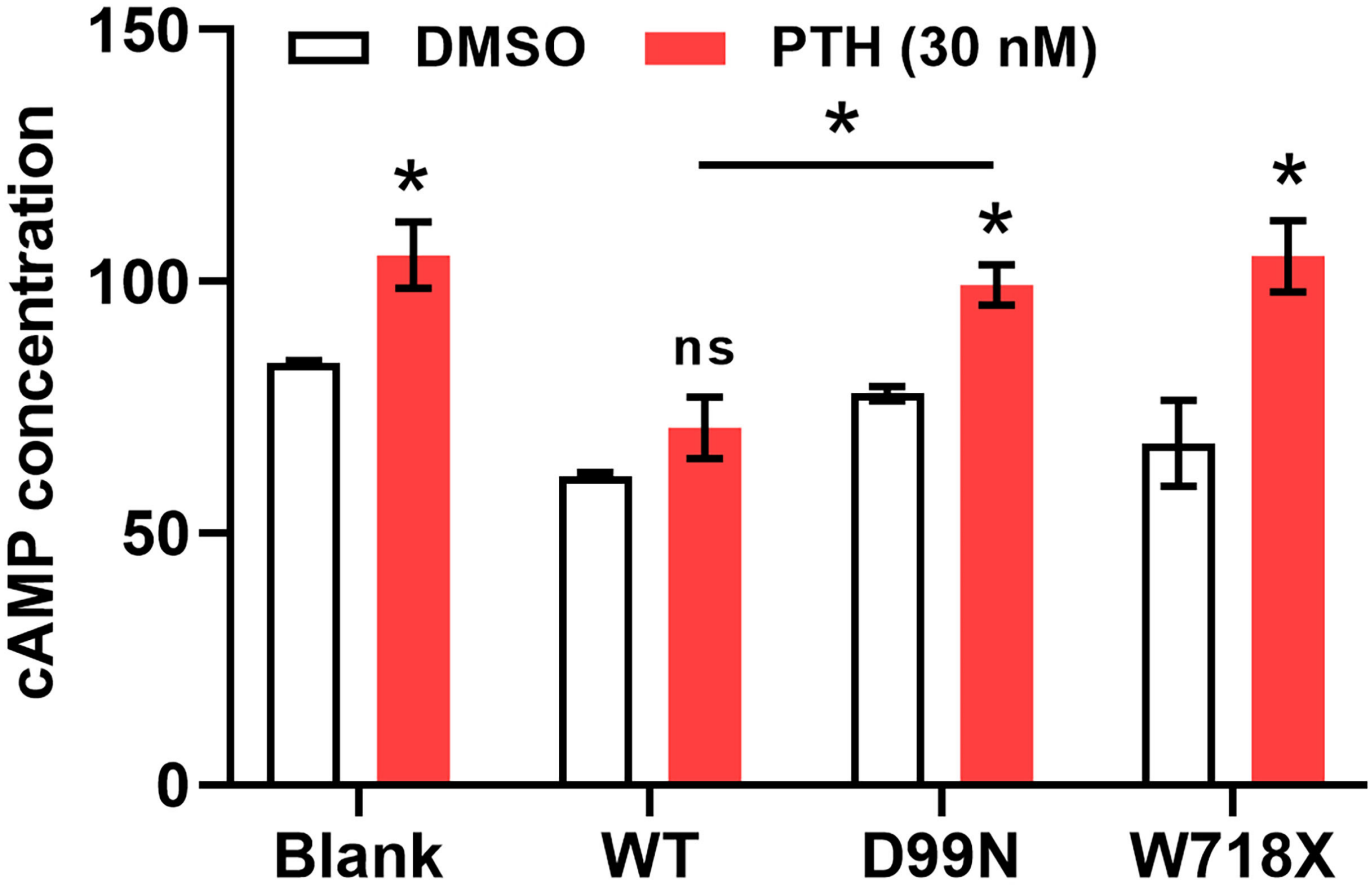
Measurement of cAMP levels in response to PTH. Intracellular cAMP levels in all groups following treatment with dimethyl sulfoxide (control) or 30 nM PTH. *P < 0.05, ns for no significance..

PTH increases cAMP levels in renal tubular cells to enhance Ca^2+^ reabsorption. We next added PTH to each group of cells to explore the effects of different CaSR gene mutations on this process by which PTH promotes calcium absorption. As shown in **Figure 6**, compared with dimethyl sulfoxide, addition of 30 nM PTH increased the intracellular cAMP levels in all groups, with significant differences within the blank group, D99N group, and W718X group (P < 0.05). Furthermore, the cAMP level in the D99N group treated with PTH was significantly greater than that in the WT group treated with PTH (P < 0.05). These results suggest that the inactivation mutation of D99N CaSR weakens the antagonizing function toward the effect of PTH.

## Discussion

The CaSR protein consists of three parts: the N-terminal extracellular domain, seven transmembrane domain, and intracellular domain (10). CaSR can detect minor changes in the serum ionized calcium concentration, and plays a key role in the regulation of calcium balance. Inactivating mutations in CaSR lead to hypocalciuric hypercalcemia, which primarily includes FHH1 and NSHPT. In addition, patients with primary hyperparathyroidism have been reported to harbor CaSR gene mutations. In a previously reported study, a patient with a parathyroid adenoma, who had not achieved clinical remission following resection of the adenoma (11), was started on a CaSR agonist that subsequently alleviated the clinical symptoms. Furthermore, statistical studies showed that the R990G polymorphism in the CaSR gene is associated with blood calcium levels (12). Therefore, the diagnosis and differential diagnosis of disorders caused by CaSR gene mutations cannot be determined based on clinical manifestations and laboratory testing alone. Additional workups involving gene sequencing and *in vitro* functional experiments must be used to guide diagnosis and treatment selection. Such approaches will also help to further our understanding of the etiology, pathogenesis, and relationships of relevant disorders.

FHH1 is an autosomal dominant genetic disease caused by heterozygous inactivating mutations in the CaSR gene, most of which are single amino acid substitutions (13). The clinical manifestations are relatively mild, most cases are identified during physical examination as mildly elevated blood calcium levels that are typically no more than 3 mmol/L, normal or slightly elevated PTH levels, and reduced urine calcium excretion. Complications of hypercalcemia are rare, and most cases of FHH1 do not require treatment. Patients with obvious symptoms may be treated with a calcimimetic such as cinacalcet. The prognosis is generally good. NSHPT is rarer than FHH1. Most NSHPTs are caused by homozygous or compound heterozygous inactivating CaSR gene mutations, but cases caused by single heterozygous mutations, such as R185Q and R227Q, have also been reported (14). Most mutations are in the ligand-binding site of CaSR. Clinically, NSHPT is manifested as prominent hypercalcemia, with blood calcium levels of 3.5–5.0 mmol/L, accompanied by increased urine calcium. It also leads to dehydration. The blood PTH levels are increased 5–10 fold, with evident PTH-associated complications such as fractures and parathyroid hyperplasia. Treatment of NSHPT focuses on the hypercalcemic crisis with the combined use of cinacalcet and bisphosphonates. If cinacalcet is not effective, total parathyroidectomy may be considered.

In the present case study, the patient harbored a GA mutation at position 295 of exon 3 in the CaSR gene that resulted in an A/A homozygous mutation of CaSR D99N with an unusual clinical feature of very high level of blood calcium (3.69 mmol/L). Amino acid 99 of CaSR lies within the extracellular Venus Flytrap module, which is the principal site of Ca^2+^ binding. Auxiliary examinations of the clinical manifestations of hypocalciuric hypercalcemia revealed that it was not completely consistent with the conventional classification of FHH1/NSHPT. Further investigations are required to expand our understanding of these disorders. For instance, the effects of the following mutations may be studied: a) mutations introduced at different positions in the CaSR protein, such as point mutations in the ligand-binding site, to investigate the direct effect on receptor function; b) mutations introduced to structures other than the binding sites to investigate how they interfere with ligand binding (15); c) point mutations introduced to extracellular and transmembrane domains to study how they affect cell surface protein expression (16); and d) mutations introduced to the intracellular C-terminus or co-expression of protein mutants may be used to study their effects on intracellular signal transduction and protein stability (17). These studies will further refine the clinical classification and aid our understanding of disorders associated with hypocalciuric hypercalcemia.

The conventional medical treatments for hypocalciuric hypercalcemia are diuresis and hydration, as well as pharmacological treatments such as calcimimetics and bisphosphonates. For refractory severe hypercalcemia, parathyroidectomy of three parathyroid glands with conservation of one parathyroid gland may be considered. In one family with hypocalciuric hypercalcemia, which was confirmed by mutational analysis to be due to an inactivating mutation in the CaSR, subtotal parathyroidectomy revealed parathyroid gland hyperplasia/adenoma and corrected the biochemical signs of the disorder in seven of nine individuals (18). Currently, parathyroid radiofrequency ablation is primarily used to treat parathyroid tumors. The use of this technique to treat a patient without a tumor has not been reported previously. The parathyroid B-mode ultrasound identified three parathyroid glands in the patient, whereas the presence of an ectopic parathyroid gland required further exploration. Ablation of the three parathyroid glands resulted in significant short-term treatment effects. The treatment outcomes remain to be evaluated in subsequent follow-ups. Additionally, further investigations are needed to delineate the extent of parathyroid radiofrequency ablation required for patients with hypocalciuric hypercalcemia caused by different types of inactivating CaSR mutations.

Taken together, the novel D99N homozygous mutation in the CaSR gene reduces CaSR activity, leading to reduced sensitivity of parathyroid cells to extracellular Ca^2+^ and abnormal PTH secretion, thereby causing more severe hypocalciuric hypercalcemia. For patients suffering from this type of severe hypercalcemia and who show a poor response to multiple pharmacological treatments, radiofrequency ablation of the parathyroid glands may be a treatment option. The specific protocol and treatment outcome remain to be investigated.

## Data Availability Statement

The original contributions presented in the study are included in the article/supplementary material. Further inquiries can be directed to the corresponding author.

## Ethics Statement

The studies involving human participants were reviewed and approved by the ethical committee of Affiliated Hangzhou First People’s Hospital, Zhejiang University School of Medicine, Hangzhou, Zhejiang province, China. The patients/participants provided their written informed consent to participate in this study.

## Author Contributions

YH conducted the experiments and wrote the manuscript. ZL performed ultrasound-guided radiofrequency ablation of the parathyroid glands. NS, LY, and WJ collected the patient’s clinical data. XZ was a major contributor in designing the study. All authors read and approved the final manuscript.

## Funding

This work was supported by Hangzhou Health Science and Technology Project of Hangzhou Municipal Health Commission [grant number 2018A08]; and Medical and Health Science and Technology Program of Health commission of Zhejiang province [grant number 2019KY487].

## Conflict of Interest

The authors declare that the research was conducted in the absence of any commercial or financial relationships that could be construed as a potential conflict of interest.

## Publisher’s Note

All claims expressed in this article are solely those of the authors and do not necessarily represent those of their affiliated organizations, or those of the publisher, the editors and the reviewers. Any product that may be evaluated in this article, or claim that may be made by its manufacturer, is not guaranteed or endorsed by the publisher.
